# Hippocampal ensembles regulate circuit-induced relapse of extinguished fear

**DOI:** 10.1038/s41380-025-03064-3

**Published:** 2025-05-24

**Authors:** James E. Hassell, Angel D. Arellano Perez, Krithika Vasudevan, Reed L. Ressler, Gabriela M. Garcia, Madison Parr, Valerie M. Vierkant, Hugo Bayer, Stephen Maren

**Affiliations:** 1 Department of Psychological and Brain Sciences and Institute for Neuroscience, Texas A&M University, College Station, TX 77843 USA; 2 Division of Endocrinology, Metabolism and Diabetes, Department of Medicine, University of Colorado Anschutz Medical Campus, Aurora, CO 80045 USA; 3 Beckman Institute for Advanced Science and Technology, University of Illinois Urbana-Champaign, Urbana, IL 61801 USA; 4 Department of Psychology, University of Illinois Urbana-Champaign, Champaign, IL 61820 USA

**Keywords:** Neuroscience, Psychology

## Abstract

Extinction learning is central to behavioral therapies for post-traumatic stress disorder (PTSD), but relapse poses a major challenge to this approach. Recent work has revealed a critical role for the thalamic nucleus reuniens (RE) in the suppression of extinguished fear memories. Silencing the RE yields a relapse of extinguished fear (i.e., “circuit-induced relapse”). Considerable work suggests that RE may contribute to extinction by inhibiting the retrieval of hippocampal (HPC)-dependent fear memories. To test this hypothesis, we first examined whether undermining the formation of contextual fear memories in the HPC would prevent circuit-induced relapse. Intra-hippocampal infusions of the NMDA receptor antagonist, APV, prior to auditory fear conditioning eliminated contextual fear memory and prevented the subsequent relapse of extinguished fear to the auditory conditioned stimulus (CS). In a second experiment, we used an activity-dependent labeling system (AAV-cFos-tTA; AAV-TRE-hM3Dq-mCherry) to express excitatory DREADDs in HPC neurons during fear conditioning. Chemogenetic reactivation of these ensembles after extinction was sufficient to drive relapse of fear to the extinguished CS. Lastly, in a third experiment, we expressed excitatory DREADDs in HPC ensembles captured during extinction learning and found that chemogenetic reactivation of this ensemble was sufficient to inhibit circuit-induced relapse. These results reveal that HPC-dependent ensembles play a critical role in regulating the expression and relapse of extinguished fear.

## Introduction

Traumatic events or intense emotional experiences can result in the formation of pathological fear memories [1]. Cognitive-behavioral therapies including prolonged exposure therapy seek to extinguish maladaptive fear responses by exposing patients to trauma reminders in a safe environment [2, 3]. Though effective, many patients are susceptible to relapse [4]. In recent years, it has been found that interactions between the medial prefrontal cortex and hippocampus regulate the expression of extinguished fear [5, 6]. For example, projections from the ventral hippocampus to the infralimbic (IL) cortex mediate the relapse or “renewal” of extinguished fear to a conditioned stimulus (CS) encountered outside the extinction context [7]. Moreover, hippocampal neuronal ensembles representing fear or extinction memories bidirectionally regulate conditioned fear responses such as freezing [8–11]. This suggests that contextual memory representations in the hippocampus may be central to the relapse of fear to an extinguished CS.

Recent work has shown the midline thalamic nucleus reuniens (RE) is critical for regulating hippocampal-prefrontal interactions central to extinction learning and retrieval [12]. For example, the RE mediates the encoding of precise memories central to the discrimination of safe and dangerous contexts [13, 14]. Moreover, pharmacological inhibition of RE or its prefrontal afferents impairs both the acquisition and retrieval of extinction memories [15–17], a phenomenon we term “circuit-induced relapse”. We have recently demonstrated that circuit-induced relapse is associated with a loss of oscillatory coherence between the hippocampus and prefrontal cortex and that theta-paced stimulation of RE principal neurons mitigates the renewal of extinguished fear [12]. Collectively, our findings support the notion that circuit-induced relapse is due to impaired mPFC-HPC coordination.

Work in both humans and animal models suggests that the RE may mediate prefrontal inhibition of hippocampus-dependent memories [18]. After extinction, we hypothesize that the RE contributes to the inhibition of conditional fear by suppressing the retrieval of contextual fear memories encoded by the hippocampus. To examine this hypothesis, we conducted three experiments to assess the role for the hippocampus in the RE-mediated (circuit-induced) relapse of extinguished fear. First, we determined whether circuit-induced relapse requires hippocampus-dependent context memories. Second, we used activity-dependent neuronal labeling methods to express excitatory “designer receptors exclusively activated by designer drugs” (DREADDs) in HPC ensembles during fear conditioning. We next determined whether chemogenetic reactivation of these ensembles during extinction retrieval caused fear relapse. Finally, after tagging hippocampal fear extinction memories using the same procedures, we determined if chemogenetic reactivation of extinction ensembles would inhibit circuit-induced relapse.

## Materials and methods

### Subjects

A total of 102 adult male and female rats (200–224 g; Long-Evans Blue Spruce; Envigo) were used in this study (Experiment 1a, *n* = 13; Experiment 1b, *n* = 37; Experiment 2, *n* = 24; Experiment 3, *n* = 28). Subjects were individually housed in a temperature-controlled and humidity-controlled vivarium, on a 14/10-h light/dark cycle, and had food and water access *ad libitum*. All experiments were performed during the light phase. The subjects were handled for 1 min every day for at least 5 days to habituate them to the experimenters.

### Viral vectors and drugs

Plasmids were a gift from the laboratory of S. Tonegawa and were packaged at the University of Pennsylvania Vector Core. Viral infusions consisted of a 50:50 viral cocktail containing AAV9.cFos.tTA9.bGH (henceforth referred to as cFos-tTA; titer: ≥ 5e13 genomic copies/mL) and AAV.TRE.hM3Dq.mCherry.rBG (henceforth referred to as TRE-hM3Dq; titer: ≥ 5e13 genomic copies/mL; Experiments 2 and 3). Muscimol (MUS; Sigma; EMD Milipore Corp.) was diluted in 0.9% sterile saline to a concentration of 0.1 µg/µL (Experiments 1 and 3). D,L-2-amino-5-phosphonovalerate (APV; Tocris Biosciences), was dissolved in 0.9% sterile saline to a concentration of 10 µg/µL (Experiment 1). Clozapine-*N*-oxide (CNO) was obtained from the Chemical Synthesis and Drug Supply Program of the National Institute of Mental Health and dissolved in 2.5% dimethyl sulfoxide (DMSO) in 0.9% sterile saline and injected systemically (3 mg/kg, i.p.). Subjects were fed a doxycycline diet (DOX, 40 mg/kg; Envigo product code TD.180316) for at least 10 d before any surgical procedures to ensure to conditionally inhibit the activity-dependent expression of hM3Dq in hippocampal neurons.

### Surgery

Subjects were anesthetized with isoflurane (5% for induction and ∼2% for maintenance; flow rate, ~0.8–1.0 L/min) and fixed with ear bars into a stereotaxic instrument (Kopf Instruments). Subjects were given eye ointment (Optixcare) and subcutaneous injections of a non-steroidal anti-inflammatory carprofen (Rimadyl, Zoetis) at a dose of 5 mg/kg. Hair on the scalp was shaved, ~250 µL of lidocaine-epinephrine (Cook-Waite) was subcutaneously injected at the future incision site, and the scalp was wiped with povidone-iodine (Medline). After a loss of the toe pinch reflex, an incision was made on the scalp to expose the top of the skull. The head was leveled [anterior/posterior (A/P) ± 0.1 mm between bregma and λ], and bregma was identified. Coordinates for craniotomy were marked on the skull and small holes were drilled into the skull to affix 2–3 jeweler’s screws. For Experiments 1a and 1b, bilateral guide cannulae targeting the dorsal hippocampus (DH) were implanted at a 20° angle from the midline (A/P: −3.7; medial/lateral (M/L): ±3.2; dorsal/ventral (D/V): −3.2 mm from skull). In Experiments 1b and 3, a single guide canula targeting the nucleus reuniens (RE) was implanted at a 10° angle from midline (A/P: −2.1; M/L: +1.25; DV: −7.09 mm from skull). For experiments that involved activity-dependent tagging (Experiments 2 and 3), infusion lines were loaded with 1 µL of viral cocktail and were lowered into the brain at a 0° angle into each hemisphere. Virus cocktail was infused at a rate of 0.1 µL/min into each of two infusion sites in the DH (A/P: −3.5 mm from bregma; M/L: ±2.5 mm; D/V: −3.2 mm for 0.5 µL and −3.1 mm for 0.5 µL (1 µL/hemisphere). Injectors were left in the brain for 10 min after both infusions in each hemisphere to allow for viral diffusion. After infusion, the injectors were slowly raised from the brain and the scalp incision was closed with suture (Experiment 2 and 3). For Experiment 1, subjects were allowed to recover for 7d after surgery before behavioral testing. For Experiments 2 and 3, subjects were given 2 weeks to allow for sufficient viral expression before behavioral testing. Guide cannulae were affixed to the skull with dental cement (CO-ORAL-ITE Dental MFC Co.), and stainless-steel dummy cannulae (P1 Tech) were inserted into the guide cannula(e). Triple antibiotic ointment (Cosette Pharmaceuticals) was applied around the headcap to prevent infection.

### Drug infusions

For Experiments 1 and 3, all rats were habituated to the microinfusion procedure prior to behavioral testing by transporting them to the infusion room and changing out dummy cannulae, keeping the context as close to the infusion context as possible, before returning them to the vivarium. For microinfusions, rats were transported in squads of four from the vivarium to an infusion room using the transport boxes, white transport boxes for context A or black transport boxes with bedding for context B. Animals were then placed from the transport boxes to white 5-gal buckets (one rat per bucket). Dummy cannulae were removed from guides, and stainless-steel injectors (33 g, 10 mm; P1 Tech) connecting polyethylene tubing (Braintree Scientific) were inserted into the guides for intracranial infusion. Internal injectors were connected to 10 µL Hamilton syringes mounted on an infusion pump (KD Scientific).

Infusions were monitored via movement of an air bubble separating water from drug in the infusion line. For RE infusions, MUS or saline was infused into the brain at a rate of 0.1 µL/min for 3 min, and the injectors remained in the brain for 3 min to allow for drug diffusion before removal. For DH infusions, APV (Tocris) or saline was also infused at a rate of 0.1 µL/min for 3 min, and injectors remained in the brain for 3 min for drug diffusion before removal. After infusions, clean dummies were secured to the guide cannulae. In Experiments 1 and 3, each rat received systemic injections of either VEH or CNO (3 mg/kg, i.p.).

### Experimental design and statistical analysis

Behavioral procedures were conducted in standard rodent conditioning chambers consisting of two aluminum walls, two Plexiglas walls, and a Plexiglas ceiling (Med Associates). Two distinct contexts were used in this study. Context A featured a 15-W house white light within each chamber, red fluorescent room lights, and 65 dB ventilation fans turned off. Chambers were positioned inside sound attenuating cabinets, with the cabinet doors closed, and the chambers were individually scented with 1% ammonium hydroxide by placing some solution in the waste pan under the grid floor unless otherwise specified. In Context B, the house lights were turned off, white, fluorescent room lights were turned on, and 65 dB ventilation fans circulated air in the cabinets. Cabinet doors were open, and black Plexiglas floors were placed atop the stainless-steel grid floor in each chamber (no shock was administered in context B). Chambers were scented with 3% acetic acid. Subjects were transported to and from context A using white transport boxes without bedding, and to and from context B using black transport boxes with fresh bedding.

Auditory stimuli were delivered by a speaker mounted on one wall of the chamber, and scrambled footshock was delivered by a grid floor composed of stainless-steel rods. The conditioned stimulus (CS) was a 10-s, 2-kHz acoustic tone and the unconditioned stimulus (US) was a 2-s, 1-mA footshock, unless indicated otherwise. Each session began with a 3-min stimulus-free baseline (BL) period. Conditioning consisted of five CS–US deliveries with a 70-s intertrial interval (ITI) and a 1-min post-trial period. Extinction consisted of 45 CS-only trials with a 40-s ITI and a 3-min post-trial period. Load-cell force transducers located underneath each chamber measured displacement of the chamber in response to motor activity; these voltages (±10 V) were acquired at 5 Hz and transformed to absolute values (scale = 0–100). A freezing bout was defined as 5 consecutive values < 10 (freezing threshold, corresponding to 1 s of freezing). Each trial was composed of the CS and ITI. For extinction sessions, 5 trials were averaged to compose a block. Extinction data were analyzed using the averages of the first block (5 trials) of the first extinction day (first) and last block (5 trials) of the last extinction day (final). Extinction retrieval consisted of 5 CS-only deliveries with a 40 s ITI and a 3 min post-trial period.

Data were analyzed with conventional parametric statistics (GraphPad Prism). Repeated-measures analysis of variance (ANOVA) was used to assess main effects and interactions (α = 0.05). For *post hoc* analyses, Fisher’s least significant difference (LSD) test was used. Results are shown as the mean ± standard error of the mean (SEM). Group sizes were determined based on our previous studies. No significant sex differences were noted in any of the analyses and data for male and female rats were therefore collapsed (the sex of the subjects is indicated by unique plot symbols in the bar graphs).

### Behavioral procedures

#### Experiment 1a

In Experiment 1a, we examined whether intra-DH APV selectively impairs contextual fear conditioning. Subjects (*n* = 13) were randomly assigned to the following groups: intra-DH vehicle (VEH) (*n* = 7) or intra-DH APV (APV) (*n* = 6). Approximately 1 week after cannula surgeries, subjects received either an infusion of VEH or APV into the DH and immediately underwent fear conditioning in context A. Subjects were returned to the vivarium after fear conditioning. 24 h after conditioning, subjects were returned to the conditioning context for a context fear retrieval test, which had a 10 min duration and did not feature any CS or US presentations. The following day, subjects were then moved to the novel context B for a CS retrieval test, during which subjects were presented with 5 CS-alone trials with 40 s ITI and pretrial and post-trial periods of 3 min each. For conditioning data, a two-way repeated-measures ANOVA was used to assess main effects (time, drug) and interactions. Extinction training was used to counterbalance groups prior to any extinction retrieval tests to ensure equivalent freezing prior to any drug. For context retrieval, and CS retrieval tests an unpaired *t*-test was performed to assess differences between drug treatment.

#### Experiment 1b

In Experiment 1b we examined whether intra-DH APV prevents RE-induced (circuit-induced) relapse. Subjects (*n* = 37) were randomly assigned to the following groups: intra-DH VEH/intra-RE VEH (VEH-VEH) or intra-DH APV/intra-RE VEH (APV-VEH); intra-DH VEH/intra-RE muscimol (VEH-MUS); or intra-DH APV/intra-RE muscimol (APV-MUS). The group sizes were as follows: VEH-VEH: *n* = 10, APV-VEH: *n* = 10, VEH-MUS: *n* = 9, APV-MUS: *n* = 8. Approximately 1 week after cannula surgeries, subjects received either an infusion of VEH or APV into the DH and immediately underwent fear conditioning in context A. Subjects were returned to the vivarium after fear conditioning. Twenty-four hours after fear conditioning, subjects underwent extinction training in context B in which subjects were presented with 45 CS-only trials. After the last day of extinction training, subjects received either intra-RE muscimol or intra-RE vehicle prior to CS retrieval test in context B, during which subjects were presented with 5 CS-alone trials with a 40-s ITI; pretrial and posttrial periods were 3 min each. All data were analyzed using ANOVA, and *post*-*hoc* comparisons in the form of Fisher’s unprotected least significant difference multiple comparisons tests were performed after a significant overall *F* ratio in the ANOVAs. All data are represented as means ± SEM.

#### Experiment 2

In Experiment 2 we examined whether chemogenetic reactivation of DH “fear” ensembles induces relapse of fear to an extinguished CS. We used an activity-dependent viral system [10, 19, 20] to express an excitatory DREADD (AAV-TRE-hM3Dq-mCherry) in DH neuronal ensembles that were active (AAV-cFos-tTA) during auditory fear conditioning. A total of 32 rats were used in this experiment and randomly assigned to each conditioning treatment and drug administration condition. Approximately two weeks after the viral infusion, subjects were taken off DOX diet to allow for neuronal tagging during fear conditioning. The day after, subjects underwent fear conditioning in context A. Subjects were returned to DOX diet immediately after fear conditioning and returned to the vivarium and remained on DOX for the remainder of the experiment to prevent further cell tagging. The experiment consisted of three groups: 1) an experimental group in which we tagged DH ensembles during conditioning and reactivated those ensembles with CNO during an extinction retrieval test (CNO), 2) a control group for which DH ensembles were tagged, except receiving VEH during the retrieval test (VEH), and 3) a control group for which DH ensembles were tagged after mere exposure to the conditioning context (no shocks were delivered) and administered CNO during the retrieval test [CNO (NoCon)]. The group sizes were as follows: CNO: *n* = 7; VEH: *n* = 9; CNO (NoCon): *n* = 8.

Forty-eight hours after tagging hippocampal ensembles during auditory fear conditioning, the rats underwent an extinction procedure in a novel context (context B). This was followed 24 h later by an extinction retrieval test in context B, during which subjects were presented with 5 CSs. Prior to the extinction retrieval test, subjects received either VEH or CNO (i.p). and were left in their home cages for thirty minutes before extinction retrieval. Extinction retrieval testing was conducted in context B and consisted of a 10-min baseline period followed by 10 CS presentations with 40-s ITIs and a 3-min posttrial period. The increased baseline duration was to see if systemic CNO had an impact on baseline freezing to context B. All data were analyzed using ANOVA, and *post*-*hoc* comparisons in the form of Uncorrected Fisher’s least significant differences multiple comparisons tests were performed after a significant overall *F* ratio in the ANOVAs. All data are represented as means + or ± SEM.

#### Experiment 3

In Experiment 3, we investigated whether chemogenetic activation of a DH extinction ensemble would inhibit RE circuit-induced relapse. To achieve this, we employed a chemogenetic strategy, as described in Experiment 2, involving the expression of the hM3Dq DREADD, and the tagging of hippocampal activity was performed during extinction retrieval. A total of 32 subjects were included in this experiment and were randomly assigned to drug manipulation and within-subject testing conditions. Initially, subjects were conditioned in Context A, where the same procedures were followed as described previously, but shocks were administered for 1 s.

The following day, extinction training was conducted in Context B. After the last day of extinction, the DOX diet was removed for 48 h to allow tagging during retrieval of extinction memory. Subjects were then returned to the DOX diet immediately after the last day of extinction. Twenty-four hours later, the animals were systematically injected with either VEH or CNO (3 mg/kg, i.p.) 30 min before being infused with either VEH or MUS into the RE. Subsequently, they underwent extinction retrieval and RE circuit-induced relapse was tested. VEH or CNO were administered on alternate days in a counterbalanced manner prior to the retrieval test after MUS or VEH infusions into RE. The final group sizes were as follows: VEH: *n* = 15; CNO: *n* = 13. A two-way repeated-measures ANOVA was used to assess main effects (drug, group) and interactions or three-way repeated-measures ANOVA (time, drug, group) and interactions.

### Histology

Upon completion of each experiment, subjects were overdosed with sodium pentobarbital (100 mg/kg, i.p.; Fatal-Plus, Vortech Pharmaceuticals) and perfused with 0.9% saline followed by 10% formalin solution. Brains were extracted and stored for 16–18 h at 4 °C in 10% formalin, after which they were transferred to a 30% sucrose solution until brains sunk to the bottom of the brain jar. Brains were embedded (OCT, Electron Microscopy Sciences, cat. no. 72592), frozen on dry ice and sectioned at 30 µm thick coronal slices using a cryostat (Leica Microsystems, model(s) CM1850 UV-3-1 or CM1860UV) at −20 °C. For imaging native fluorescence of tagged cells in the DH, brain slices were dry mounted onto microscope slides and coverslipped with Fluoromount mounting medium (Invitrogen).

To identify cannula placements, brain slices were dry mounted onto gelatin-subbed slides (Fisher scientific, cat. no. 125442) and stained with thionin. Specifically, tissue slides were submerged for 5 min each in 95 and 100% ethanol, followed by 10 min of submersion in CitriSolv (Fisher Scientific). Mounted tissue was then submerged in 100% ethanol (3 min), 95% ethanol (2 min), 70% ethanol (2 min) followed by 0.25% thionin for ~20 s. Mounted tissue was then rinsed in distilled water followed by submersion in 70% ethanol and 0.01% acetic acid (1 min), 70% ethanol (1 min), 95% ethanol (4 min), and 100% ethanol (4 min) before submersion in CitriSolv for 10 min before cover slipping. Glass coverslips (Avantor, cat. no. 48393-251) were mounted on slides using Permount mounting media (Fisher Scientific, cat. no. SP15-500). Thionin stained coronal sections were imaged using a Leica Microscope (MZFLIII) with Leica Firecam software and native fluorescent mCherry were imaged using a Zeiss microscope with a Plan-Apochromat 10x/0.45 NA objective (Zeiss), AxioCam Mrm camera, and Axio Imager software (Zen Pro 2012).

## Results

### Experiment 1: Hippocampal context memory is necessary for circuit-induced relapse

To determine if hippocampus-dependent contextual memories are required for circuit-induced relapse, we assessed whether preventing the encoding of contextual fear memories would attenuate RE-induced relapse. As a first step, we assessed whether intra-hippocampal infusions of D,L-2-amino-5-phosphonovaleric acid (APV), an NMDA receptor antagonist, would selectively attenuate contextual fear conditioning [13, 21, 22].

**Fig. 1:**
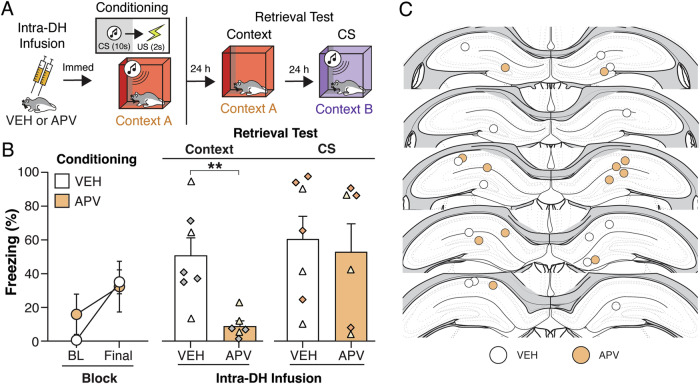
Dorsal hippocampal fear memory is specific to context and not conditioned stimuli (CS). **A** Experimental design of adult male and female rats that were surgically implanted with cannulae aimed at the dorsal hippocampus (DH). After recovery, animals were infused with vehicle (VEH) or the *N*-methyl-D-aspartate (NMDA) receptor antagonist, D,L-2-amino-5-phosphonovalerate (APV), just prior to auditory fear conditioning (VEH, *n* = 7; APV, *n* = 6). After conditioning, animals underwent a context retrieval test followed 24 h later by an auditory conditioned (CS) retrieval test. **B** *Left panel* displays percentage of freezing [mean ± standard error of the mean (SEM)] during the baseline (BL) and final conditioning CS. *Right panel* displays percentage of freezing (mean ± SEM) during the context and tone retrieval. Female animals are represented by white-filled and light orange-filled triangles, while male animals are represented by gray-filled and dark orange-filled diamonds in the bar graphs. **p < 0.01. **C** Representative location of the injector tip of the cannula tract within the DH, of each animal included in the final analyses. Atlas figures are adapted from Swanson (2018).

As illustrated in Fig. 1A, rats received bilateral infusions of APV into the dorsal hippocampus immediately prior to auditory fear conditioning in a novel context. Two subjects were excluded from the statistical analysis: one for an incorrect cannula placement and the other for technical issues in tissue processing that prevented assessment of cannula placement. After placement in the conditioning context, rats showed low levels of baseline (BL) freezing prior to tone presentation (Fig. 1B, left panel). Once conditioning commenced, freezing increased across the conditioning session (Fig. 1B, left panel). This observation was confirmed in a two-way repeated measures ANOVA which revealed a main effect of trial [*F*_(1, 11)_ = 17.91 ; *p* = 0.0014] with no main effect of group [*F*_(1, 11)_ = 0.2626; *p* = 0.6185].

Twenty-four and 48 h after fear conditioning, respectively, rats underwent drug-free context (10 min) and CS retrieval (5 CS-alone trials) sessions (Fig. 1B, right panel). During the context retrieval session, there was a main effect of drug (*t*_(11)_ = 3.606; *p* = 0.0041), with APV-treated rats freezing significantly less than vehicle controls. In contrast, there was no main effect of APV-treatment on conditioned freezing during the CS retrieval session (*t*_(20)_ < 1.0; *p* = 0.7286). A schematic illustrating cannulae placements with the DH is shown in Fig. 1C.

**Fig. 2:**
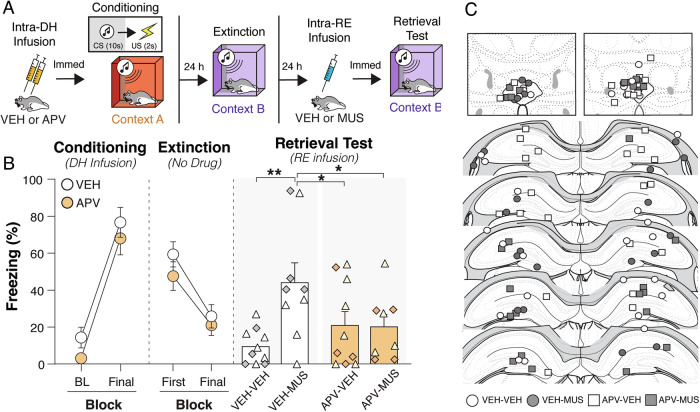
Circuit-induced relapse requires dorsal hippocampal fear memory. **A** Experimental design of adult male and female rats that were surgically implanted with cannulae aimed at the dorsal hippocampus (DH) and nucleus reuniens (RE). After recovery, animals were infused with vehicle (VEH) or the *N*-methyl-D-aspartate (NMDA) receptor antagonist, D,L-2-amino-5-phosphonovalerate (APV), just prior to auditory fear conditioning (VEH, *n* = 19; APV, *n* = 18). After conditioning, animals underwent auditory CS extinction. After the last extinction session, animals received VEH or the γ-amino butyric acid (GABA)_A_ receptor agonist, muscimol (MUS), just prior to auditory extinction retrieval giving four groups (VEH-VEH, *n* = 10; APV-VEH, *n* = 10; VEH-MUS, *n* = 9; APV-MUS, *n* = 8). **B** *Left panel* displays percentage of freezing [mean ± standard error of the mean (SEM)] during the baseline (BL) and final conditioning CS. *Middle panel* displays percentage of freezing (mean ± SEM) during the first extinction block and the final extinction block. *Right panel* displays percentage of freezing (mean + SEM) with animals either receiving intra-RE VEH or MUS prior to extinction retrieval. Female animals are represented by white-filled and light orange-filled triangles, while male animals are represented by gray-filled and dark orange-filled diamonds in the bar graphs. *p < 0.05; **p < 0.01. **C** Representative location of the injector tip of the cannula tract in RE, *top row*, or DH, *bottom row*, of each animal included in the final analyses. Atlas figures are adapted from Swanson (2018).

We next asked whether circuit-induced relapse would occur in rats that had been conditioned after APV infusions into the DH. In other words, does circuit-induced relapse require a contextual fear memory? As in the previous experiment, rats received bilateral DH infusions of APV or vehicle immediately prior to auditory fear conditioning (Fig. 2A). The next day, extinction to the auditory CS was conducted in a novel context. Twenty-four hours after extinction, rats were infused with intra-RE muscimol (0.1 µg µL^−1^; 0.3 µL total; 0.1 µL min^−1^) immediately prior to an extinction retrieval test. Five rats were excluded from the statistical analysis due to incorrect cannula placement. As shown in Fig. 2B (left panel), there was no main effect of group on freezing behavior during conditioning [*F*_(1, 35)_ = 0.6061; *p* = 0.4432]; a main effect of trial [*F*_(1, 35)_ = 118.0; *p* < 0.0001] revealed that rats successfully acquired fear memory across the conditioning session (Fig. 2B, left panel). During fear extinction (Fig. 2B, middle panel), there was also a main effect of trial [*F*_(1,35)_ = 39.71; *p* < 0.0001] but no main effect of group [*F*_(1, 35)_ = 1.680; *p* = 0.2034].

Prior to the extinction retrieval test, rats received intra-RE infusions of either muscimol or saline, counterbalanced for prior APV treatment. As shown in Fig. 2B (right panel), muscimol infusions into the RE caused a relapse of conditioned freezing to the extinguished CS in VEH- but not APV-treated rats. In other words, rats conditioned after DH infusions of APV failed to exhibit circuit-induced relapse. This observation was confirmed by a significant interaction of HPC (VEH or APV) and RE (VEH or MUS) drug treatments [*F*_(1, 33)_ = 6.021; *p* = 0.0196]; there was no main effect of DH drug treatment [*F*_(1, 33)_ = 0.752; *p* = 0.392], but there was a main effect of RE muscimol [*F*_(1, 33)_ = 5.552; *p* = 0.0245]. Post-hoc comparisons revealed that RE muscimol infusions yielded a significant increase in freezing relative to RE vehicle infusions only in rats trained with VEH infusions in the DH [*t*_(33)_ = 3.457; *p* = 0.0015], a result which is consistent with previous work in our laboratory [16, 23]. Rats that were treated with DH-APV prior to fear conditioning did not exhibit MUS-induced relapse (Fig. 2B, right panel). These data strongly suggest that rats must have an intact hippocampus-dependent contextual fear memory to show relapse of fear to an extinguished CS. A schematic illustrating cannulae placements with the RE and DH is shown in Fig. 2C.

### Experiment 2: Chemogenetic reactivation of a hippocampal fear ensemble drives relapse of extinguished fear

**Fig. 3:**
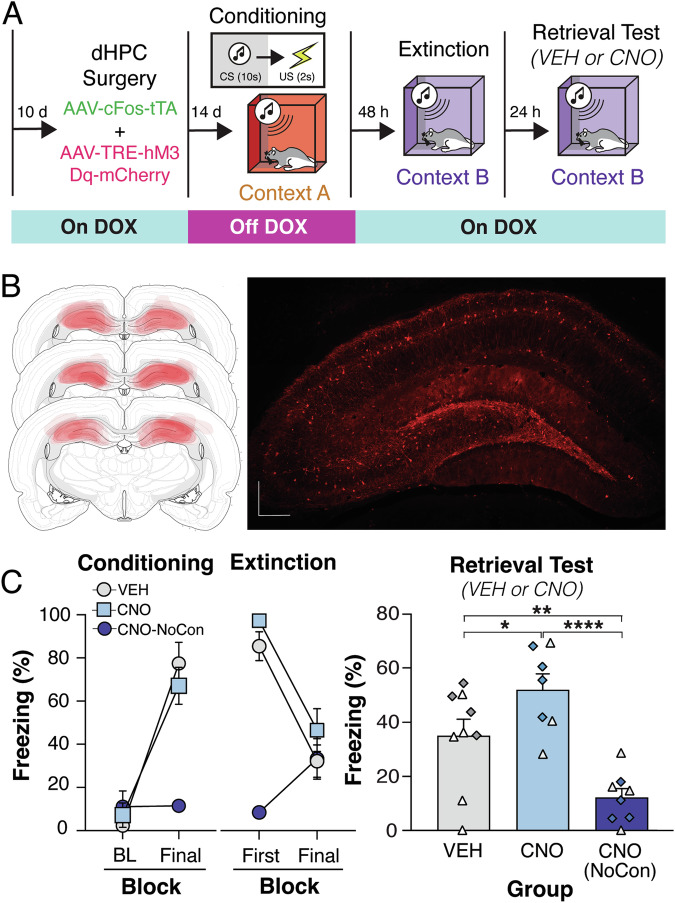
Chemogenetic reactivation of hippocampal fear ensembles drives relapse of extinguished fear. **A** Experimental design of adult male and female rats that were placed on doxycycline (DOX) diet and received cFos-tTA and TRE-hM3Dq viruses aimed at the dorsal hippocampus (DH). After recovery, all animals were removed from the DOX diet. Forty-eight hours later, two groups of animals underwent auditory fear conditioning; a third group was simply exposed to the conditioning chamber (VEH, *n* = 9; CNO, *n* = 7; CNO-NoCon, *n* = 8). After conditioning, animals were returned to DOX diet, and 48 h later underwent extinction (45 CS-alone trials). Twenty-four hours after the final extinction session, animals received the designer drug clozapine-*N*-oxide (CNO), immediately prior to an extinction retrieval test. **B** *Left panel* Schematic representation of viral expression within the DH for all the animals (viral expression common to all rats is indicated by the most darkly shaded areas). *Right panel* Representative immunofluorescent image of cFos-tTA and TRE-hM3Dq mCherry expression in the DH. Atlas figures are adapted from Swanson (2018). **C** *Left panel* displays percentage of freezing [mean ± standard error of the mean (SEM)] during the baseline (BL) and final conditioning CS. *Middle panel* displays percentage of freezing (mean ± SEM) during the first extinction block and the final extinction block. *Right panel* displays percentage of freezing (mean + SEM) with animals either receiving intra-RE VEH or MUS prior to extinction retrieval. Female animals are represented by white-filled, light gray-filled, and light blue-filled triangles, while male animals are represented by dark gray-filled, light blue-filled, and dark blue-filled diamonds in the bar graphs. *p < 0.05; **p < 0.01; ****p < 0.0001.

The previous experiment reveals that hippocampal-dependent contextual fear memories are necessary for the relapse of fear to an extinguished CS. In this experiment, we determined whether these representations are sufficient for the relapse of extinguished fear. To this end, we utilized an activity-dependent neuronal tagging method to express an excitatory DREADD in hippocampal neurons active during fear conditioning [10, 19, 20]. As illustrated in Fig. 3, we infused a cocktail of AAV-cFos-tTA and AAV-TRE-hM3Dq virus into the DH as previously described [10]. AAV-cFos-tTA results in the expression of tetracycline transactivator (tTA) in active neurons (indexed by c-Fos expression). However, when DOX is present, tTA is unable to activate the tetracycline response element (TRE) promoter on the DREADD virus (AAV-TRE-hM3Dq). When rats are removed from the DOX diet, tTA binds to the TRE promoter and drives hM3Dq expression. Rats were continuously fed DOX prior to surgery to limit activity-dependent tagging of cells before fear conditioning and were removed from DOX 24 h before fear conditioning. Seven rats were excluded from the statistical analysis based on behavioral criteria. These rats either failed to condition (*n* = 5; average freezing of at least 33%) or failed to extinguish (*n* = 2; >80% freezing in the final block).

During fear conditioning, subjects exhibited low levels of baseline freezing prior to the first trial and freezing increased across the session after the conditioning trials (Fig. 3C, left panel). This observation was confirmed with a two-way repeated measures ANOVA that revealed a main effect of conditioning block [*F*_(1, 21)_ = 113.4; *p* < 0.0001] and a main effect of group [*F*_(2, 21)_ = 6.279; *p* < 0.0152] with CNO (NoCon) showing little freezing. After fear conditioning, the subjects were returned to a DOX diet. Forty-eight hours after conditioning, subjects underwent extinction. Subjects that were fear conditioned gradually decreased freezing across the course of the extinction session (Fig. 3C, right panel). This was confirmed by a main effect of trial [*F*_(1, 21)_ = 30.20, *p* < 0.0001]. There was also a main effect of group [*F*_(2, 21)_ = 20.30, *p* < 0.0001]; rats that were fear conditioned and those that were not. In contrast to the conditioned rats, CNO (NoCon) rats exhibited a significant increase in freezing from the first to final extinction block [*t*_(21)_ = 2.993; *p* = 0.0069]; this likely reflects a general decrease in locomotor activity over the course of the extinction session.

Twenty-four hours after extinction training, rats underwent an extinction retrieval test. Thirty minutes before testing half of the fear-conditioned rats received systemic injections of VEH or CNO; CNO (NoCon) rats received CNO injections to index the consequence of reactivation of a DH ensemble tagged in the absence of shock. During the 10-min pre-CS baseline period, there were no group differences in conditioned freezing [*F*_(18,189)_ = 0.5788; *p* = 0.9119]. Under these conditions, chemogenetic reactivation of the conditioning ensemble was not sufficient to increase freezing in the extinction context (perhaps because the extinction context acquires inhibitory properties). However, as shown in Fig. 3C, average freezing during the CS trials was significantly different between the groups [*F*_(2,21)_ = 13.30; *p* = 0.0002]. Freezing was greater in CNO-treated rats relative to VEH-treated controls (or non-conditioned rats that received CNO). This was confirmed in *post*-*hoc* comparisons that revealed that COND-CNO rats showed more freezing during the test than COND-VEH rats [*t*_(21)_ = 2.244; *p* = 0.0357]. This indicates that chemogenetic reactivation of the conditioning ensemble was sufficient to drive relapse of fear to the extinguished CS without increases freezing to the context alone. A schematic illustrating cannulae placements with the DH is shown in Fig. 3B.

### Experiment 3: Chemogenetic reactivation of a hippocampal ‘extinction’ ensemble inhibits circuit-induced relapse of extinguished fear

**Fig. 4:**
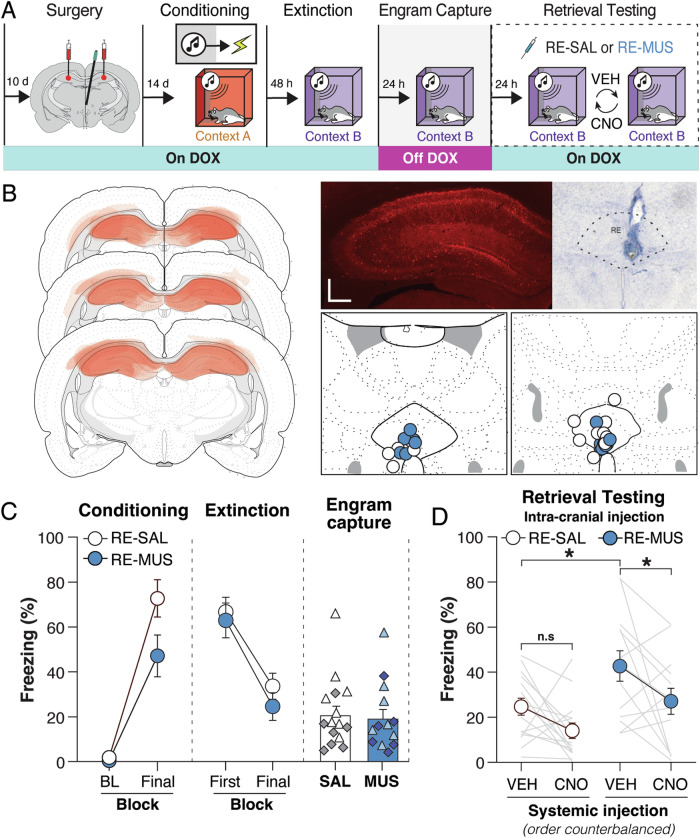
Chemogenetic reactivation of a hippocampal extinction engram inhibits circuit-induced relapse of extinguished fear. **A** Adult male and female rats were placed on doxycycline (DOX) diet and injected with AAV-cFos-tTA and AAV-TRE-hM3Dq into the dorsal hippocampus (DH); a guide cannula was implanted into the nucleus reuniens (RE). After recovery from surgery, all animals underwent auditory fear conditioning. After conditioning, animals underwent extinction. After the last extinction session, animals were removed from the DOX diet and underwent an extinction retrieval session to capture DH extinction ensembles (“engram capture”). After engram capture, animals were returned on DOX chow diet for the remainder of the experiment. Retrieval testing consisted of two counterbalanced sessions to assess whether reactivation of the extinction engram affects circuit-induced relapse. Prior to the retrieval tests, animals received intra-RE infusions of either saline (SAL) or muscimol (MUS)—this drug assignment did not change for the two tests. In addition, animals received a systemic (i.p.) injection of either vehicle (VEH) or the designer drug clozapine-*N*-oxide (CNO); each animal was injected with these drugs in a counterbalanced manner across the two retrieval tests. **B** *Left panel*, Schematic representation of viral expression within the DH for all the animals (viral expression common to all rats is indicated by the most darkly shaded areas). *Left panel*, *top row*, representative immunofluorescent image of AAV-cFos-tTA and AAV-TRE-hM3Dq mCherry expression in the DH, *Right panel*, *top row* representative thionin-stained coronal section showing a midline cannula placement in the RE, *Right panel*, *bottom row* Cannula placements for all rats that were included in the analysis. Atlas figures are adapted from Swanson (2018). **C** *Left panel* displays percentage of freezing [mean ± standard error of the mean (SEM)] during fear conditioning during the baseline (BL) period and final CS. The group labels indicate the intracranial drug assignments for subsequent retrieval tests [saline (RE-SAL), *n* = 15; muscimol (RE-MUS), *n* = 13).; *middle panel*, displays percentage of freezing (mean ± SEM) during the extinction session for the first 5-trial block and the final extinction 5-trial block; *right panel* displays the percentage freezing (mean + SEM) for the engram capture session. White-filled and light blue-filled triangles represent female animals, while dark gray-filled and dark blue-filled diamonds represent male animals in the bar graph. **D** Percentage of freezing (mean ± SEM) for the extinction retrieval test, animals received either VEH or CNO injections, in a counterbalanced manner, to reactivate the DH extinction engram. Systemic CNO decreased freezing in animals receiving either SAL or MUS infusions into the RE thereby reducing the magnitude of circuit-induced relapse. *p < 0.05; n.s. = not significant.

Our previous results demonstrated that fear memory in the DH is necessary for circuit-induced relapse, and that chemogenetic reactivation of the hippocampal fear memory engram is sufficient for relapse. Here, we aimed to investigate whether the activation of a hippocampal extinction ensemble would mitigate circuit-induced relapse. To address this question, we employed the same viral strategy as described previously to tag the hippocampal ensemble during the retrieval of an extinction memory (Fig. 4A).

During conditioning, all the rats exhibited low baseline levels; freezing increased once conditioning commenced (Fig. 4C, left panel). This observation was confirmed by a two-way repeated measures ANOVA which revealed a main effect of block [*F*_(1, 26)_ = 91.52; *p* < 0.0001]. The next day, rats were placed in context B for an extinction session consisting of 45 CS-alone trials. During the extinction session (Fig. 4C, middle panel) freezing across the groups did not differ [*F*_(1, 26)_ = 0.009, *p* = 0.927]. Conditioned freezing decreased across the extinction session [*F*_(1, 26)_ = 63.00; *p* < 0.0001]. Immediately after the last extinction session, animals were removed from the DOX diet to open the window for tagging hippocampal activity during an extinction retrieval session. Subjects were returned to the DOX diet once the retrieval session ended.

After capturing the DH extinction ensemble, we asked whether reactivation of this ensemble would decrease circuit-induced relapse produced by pharmacological inactivation of the RE. Twenty-four hours after tagging the extinction ensemble, rats received VEH or CNO injections (counterbalanced) prior to an extinction retrieval session in which animals were undergoing circuit-induced relapse. For this purpose, animals received intra-RE muscimol (0.1 µg/µL at 0.1 µL/min; 0.3 µg total) or saline immediately prior to the extinction retrieval test. A schematic illustrating viral expression within the DH and cannulae placements with the RE is shown in Fig. 4B. After testing, DREADD expression was assessed. One rat in the CNO group was excluded due to a lack of mCherry expression in the hippocampus, two rats had misplaced RE cannulas, and another rat was excluded for technical reasons.

As shown in Fig. 4D, muscimol infusions into the RE caused a relapse of freezing to the extinguished CS as we have previously reported. This observation was confirmed in a repeated measures ANOVA which revealed a main effect of RE drug treatment [*F*_(1,26)_ = 10.31; *p* = 0.0035]. Importantly, however, chemogenetic reactivation of the extinction ensemble (in rats receiving systemic CNO administration) reduced fear relapse. This was confirmed in the ANOVA by a three-way interaction of trial of test minute and the two drug manipulations [*F*_(5,130)_ = 2.491; *p* = 0.0344]. *Post hoc* comparisons revealed significant differences in the muscimol-treated animals receiving VEH or CNO [*t*_(26)_ = 2.301; *p* = 0.0297]. Hence, chemogenetic reactivation of the hippocampal extinction ensemble attenuated circuit-induced relapse.

## Discussion

In the present experiments, we show the hippocampus plays a critical role in the relapse of extinguished fear, including circuit-induced relapse (i.e., the relapse of extinguished fear that occurs after pharmacological inactivation of the nucleus reuniens). First, animals who fail to acquire a contextual fear memory after intra-hippocampal APV infusions, fail to show circuit-induced relapse. Second, chemogenetic reactivation of a hippocampal “fear” ensemble was sufficient to drive the relapse of extinguished fear. Finally, circuit-induced relapse was attenuated by reactivating a hippocampal “safety” ensemble. Together, these results indicate that both circuit-induced relapse and the expression of extinction require hippocampus-dependent neuronal ensembles. Ultimately, the RE appears to regulate the context-dependent expression of extinguished fear by both inhibiting the expression of context-inappropriate fear memories and facilitating the retrieval of extinction memories encoded in the hippocampus.

It is well established that the hippocampus is critical for the formation of long-term memory contextual fear memories [24–26]. Consistent with this work, we find that intra-hippocampal APV infusions selectively impaired contextual fear conditioning and spared conditioned freezing to an auditory CS. This is consistent with selective role for hippocampal NMDA receptors in contextual fear conditioning reported by others [27–29]. Critically, we now show that animals that fail to acquire a hippocampus-dependent contextual fear memory, fail to exhibit circuit-induced relapse of extinguished fear. That is, rats receiving intra-hippocampal APV infusions before conditioning, and subsequently extinguished, showed no increase in conditioned freezing to the extinguished CS after pharmacological inactivation of the RE. This result clearly indicates that the formation of hippocampus-dependent contextual fear memories is necessary for the relapse of extinguished fear that occurs when the RE is silenced. The hippocampus is also required for other forms of relapse, including renewal of extinguished fear that occurs when an extinguished CS is presented outside of the extinction context [30, 31]. Interestingly, the present work suggests that contextual fear memories encoded by the hippocampus may serve to promote fear relapse under some conditions. This is consistent with behavioral work showing that the conditioning context can act like a positive occasion setter by promoting the renewal of freezing to an extinguished CS [32]. Further work is required to understand, however, whether manipulations that attenuate the formation of hippocampus-dependent contextual memory (such as intra-hippocampal APV infusions) impair renewal in the same way that they impair circuit-induced relapse.

Work in humans demonstrates that the prefrontal cortex interacts with the hippocampus to inhibit the retrieval of context-inappropriate memories; this contributes to active forgetting and retrieval suppression during episodic memory retrieval [18]. The RE may mediate these processes by regulating the synchronization of prefrontal-hippocampal networks during memory retrieval [12]. Our findings demonstrate that preventing the formation of contextual fear can mitigate RE circuit-induced relapse. Consistent with this, previous work shows that pharmacological inactivation of the HPC reduces the renewal of fear to an extinguished CS when tested outside the extinction context [31, 33–35]. Collectively, this work suggests that the relapse of extinguished fear may involve the inappropriate retrieval of hippocampus-dependent fear memories. Prefrontal cortical projections to the RE may serve to inhibit the retrieval of context-inappropriate memories to prevent the relapse of fear in the extinction context. Interestingly, the suppression of fear memories in the hippocampus might be achieved through the retrieval of inhibitory extinction memories that directly compete with fear memory retrieval within distinct hippocampal ensembles [8, 23].

The RE interacts with the hippocampus not only during memory retrieval, but also during the encoding of contextual fear memories [13, 14]. An interesting question is whether the critical role for the RE in encoding extinction memories [15, 16] also requires hippocampus-dependent fear memories. Based on the present work, we would predict that preventing the formation of hippocampus-dependent fear memory (as in Experiment 1) would enable fear extinction under RE inactivation. In other words, extinction learning may become independent of the prefrontal-RE circuit if fear conditioning is acquired in the absence of a hippocampus-dependent fear memory. Overall, the RE may be critical for integrating hippocampal context information in a way that promotes context-appropriate behaviors during both fear conditioning and extinction.

Previous work in mice has shown that optogenetic activation of hippocampal neurons tagged during contextual fear conditioning can cause a relapse of contextual freezing after extinction [8]. We now show in rats (Experiment 2) that chemogenetic reactivation of hippocampal fear ensembles can also drive relapse of fear to an extinguished auditory CS. Notably, reactivation of tagged ensembles in this case did not increase freezing behavior in the extinction context (i.e., there was no increase in baseline freezing prior to delivery of the extinguished CS). These data suggest that contextual representations in the DH not only modulate the expression of conditioned freezing to a shock-associated context [8, 36] but also can influence conditioned freezing to an extinguished auditory CS. This suggests that hippocampal ensembles tagged during auditory fear conditioning might represent not only the context-US association but also represent both contextual representations and context-CS associations that can regulate freezing to a discrete CS [30]. Together, these studies suggest that even though the DH is not necessary for conditioned fear to an auditory CS ([37, 38]; but see [39]), DH engrams modulate the context-dependent retrieval of these cues. This work suggests that hippocampal ensembles tagged during fear conditioning can serve as occasion setters [30, 40, 41]. From this perspective, chemogenetic activation of the conditioning-tagged ensemble may not be sufficient to drive conditioned freezing, particularly in a potentially inhibitory extinction context. However, when reactivated in the presence of the CS, we observed that conditioned freezing to the CS is increased. This suggests that the occasion setting properties of the hippocampal ensemble may be critical for driving the relapse of fear to an extinguished CS.

The inability of chemogenetic reactivation to increase freezing in the extinction context (before the CS is delivered) might also be due to the potentially inhibitory properties of the test situation [42, 43]. Presentation of an excitatory CS without a US would be expected to foster conditioned inhibition by the extinction context. In other words, the extinction context comes to signal when the CS is safe, and therefore itself becomes an inhibitor. Several studies provide evidence that the inhibitory properties of contexts that have hosted extinction cannot by themselves account for the suppression of fear to an extinguished CS [30]. However, it is possible that the inhibition accrued by an extinction context is sufficient to reduce freezing associated with artificial reactivation of excitatory engrams. Further work is required to explore this possibility.

The relapse of extinguished fear produced by chemogenetic reactivation of hippocampal fear ensembles might have resulted from a perceived change in context associated with the reactivation manipulation. That is, reactivating the conditioning ensemble in the extinction context might have been functionally equivalent to transporting the rat to a novel context that was neither the conditioning nor extinction context. Of course, it is well known that extinguished fear renews outside the extinction context [44–47]. However, Experiment 3 demonstrated that chemogenetic reactivation of an extinction-tagged ensemble caused a reduction in relapse. If artificial reactivation of hippocampal representations was equivalent to placing the animals in a novel context, one would have expected relapse to be exacerbated. The fact that relapse was mitigated by ensemble reactivation in Experiment 3 suggests that a chemogenetic context shift cannot account for our results.

In addition to driving relapse of extinguished fear, we found that reactivating extinction-tagged ensembles in the hippocampus attenuates circuit-induced relapse. This has parallels to work in mice that showed reactivating hippocampal ensembles tagged in an extinguished context can suppress the spontaneous recovery of fear [8]. The RE may enable extinction by inhibiting hippocampal ensembles encoding the contextual fear memory [48, 49]. Because we have shown that circuit- induced relapse involves HPC-PFC interactions [12], these results further suggest that the RE may arbitrate prefrontal-hippocampal interactions involved in not only the suppression of hippocampus-dependent contextual fear memories, but also in the retrieval of extinction memories. In Experiment 3, chemogenetic reactivation of the hippocampal extinction engram mitigated circuit-induced relapse suggesting that RE might normally suppress fear by regulating both the retrieval of both fear and extinction memories.

In conclusion, we have shown that hippocampus-dependent contextual memories are critical for the regulation of fear to an extinguished CS, and circuit-induced relapse of fear after inactivation of the RE. Relapse of fear to an extinguished CS does not occur in the absence of a hippocampus-dependent contextual fear memory, chemogenetic reactivation of a hippocampal fear ensemble is sufficient to drive relapse to an extinguished CS, and chemogenetic reactivation of a hippocampal extinction ensemble mitigates circuit-induced relapse. Our data support the idea that the RE is a critical hub for PFC-HPC interactions that regulate the context-dependent retrieval of aversive memories. Future work is required to determine whether the principles described here extend to other behaviorally relevant forms of relapse.

## Data Availability

The datasets generated and analyzed during this study are available from the corresponding author upon request.
